# Prevalence of Undernutrition and Associated Factors among Lactating Mothers of Angecha District, Kembata Tembaro Zone, Southern Ethiopia

**DOI:** 10.1155/2021/6691982

**Published:** 2021-04-27

**Authors:** Moges Muluneh Boke, Aman Yesuf, Befikadu Tariku Gutema

**Affiliations:** ^1^University of Gondar School of Public Health, Gondar, Ethiopia; ^2^St. Paul's Hospital Millennium Medical College, Addis Ababa, Ethiopia; ^3^School of Public Health, Arba Minch University, Arba Minch, Ethiopia

## Abstract

**Background:**

Major reasons for malnutrition, particularly among those who live in low- and middle-income countries, are physiological vulnerability and inadequate intake. The objective of the study was to assess the prevalence of undernutrition and associated factors among lactating mothers of Angecha District, Southern Ethiopia.

**Methods:**

A community-based cross-sectional study was conducted among randomly selected lactating mothers in Angecha District from March to April 2017. A pretested structured questionnaire was used to assess the prevalence of undernutrition and associated factors among lactating mothers. Undernutrition was defined as the body mass index of less than 18.5 kg/m^2^. A multivariable logistic regression model was fitted, and the adjusted odds ratio (AOR) at a *p* value less than 0.05 was used to determine a statistically significant association between predictors and outcome variables.

**Result:**

The prevalence of undernutrition among lactating mothers was 21.2% (95% CI: 17.52, 25.46). The odds of undernutrition were higher among lactating mothers in the younger age group (AOR 4.12 (95% CI: 1.25–13.63), compared to 36–49 years group), dietary diversity less than five food groups (AOR 2.4, 95% CI: 1.35–4.36), and not attending antenatal care (ANC) (AOR 2.90 (95% CI: 1.43–5.86), compared to those who attended ANC for 4 or more times). The odds of undernutrition among lactating women from 3^rd^ quantile wealth index households reduced by nearly half (AOR 0.47, 95% CI: 0.23–0.98) compared to lactating mothers from 1^st^ quantile wealth index households.

**Conclusion:**

Nearly one in every five lactating mothers was undernourished. Age, dietary diversity score, ANC visit, and wealth index were found to be the associated factors of undernutrition. Therefore, due attention should have to be given to increase the use of ANC.

## 1. Background

Nutrition has a fundamental role in human life, health, and development throughout the entire lifespan, and its deficiency is prevalent in many countries [1, 2]. In low- and middle-income countries, maternal and child undernutrition related mortality and morbidity remain inescapable. Undernutrition is the underlying cause of 20% of maternal death and prevalent among lactating women in many regions of the world. In most countries, maternal undernutrition (BMI less than 18.5 kg/m^2^) ranges from 10% to 19% [2–4]. A serious problem of maternal undernutrition was marked in most Sub-Saharan Africa countries, where two in every 10 women were undernourished [5]. Similarly, a report based on Ethiopian DHS 2016 showed that two in every ten women suffered from undernutrition in Ethiopia [6].

Maternal nutrition is important to child survival and development [7]. Lactating mothers' nutritional status and intake determine some of the nutritional components of breast milk and child nutritional status as evidence revealed that nutrient content, specifically the concentrations of fatty acids, iodine, fat, vitamin A, thiamin, and cobalamin, depends on corresponding dietary intakes of these nutrients in the maternal diet and lactating women's nutritional status [8–11]. Inadequate intake of food and nutrients risks maternal depletion. Therefore, lactating mothers have to increase their intake to prevent maternal depletion and to produce adequate milk for the infant [7, 12, 13]. The major reasons for women's malnutrition, particularly among those who live in low- and middle-income countries, are physiological vulnerability and inadequate intake of food [14].

Ethiopia has been implementing different policies and programs including the expansion of health and nutrition services to the community. Moreover, lactating mothers' undernutrition remains a persistent problem in low- and middle-income countries including Ethiopia due to different reasons [5, 6]. The National Nutritional Strategy and Program of Ethiopia considers lactating mothers as a vulnerable group for malnutrition and gives priorities for nutrition related intervention. One of the initiatives of the program is to improve the nutrition of pregnant and lactating women [15, 16]. The Seqota declaration, a commitment by the Government of Ethiopia to end child undernutrition in Ethiopia by 2030, considers pregnant and lactating mothers as a target group for the implementation [17]. Despite the implementation of the interventions, evidence related to lactating mothers' undernutrition is limited in Ethiopia. Therefore, the aim of the study was to assess the prevalence of undernutrition and associated factors among lactating mothers of Angecha District, Southern Ethiopia.

## 2. Methods and Materials

### 2.1. Study Setting and Area

The study was conducted in Angecha District, Kembata Tembaro Zone, Southern Ethiopia. The administrative center of the district is Angecha town, which is located 255 km to the South of Addis Ababa, the capital city of Ethiopia. According to the Central Statistical Agency population projection for Ethiopia, in 2016, the total population of the district was 94,978 [18] and the district health office report showed that there are 20 health posts and 5 health centers.

### 2.2. Study Design, Period, and Population

A community-based cross-sectional study was conducted from March to April 2017. The inclusion criteria for the study population were lactating mothers whose child's age was less than 2 years and who lived in the study area for more than 6 months. Based on the review of the health posts' record, 2,825 eligible lactating women were identified.

### 2.3. Sample Size Determination and Procedures

To determine the sample size of the study, single population proportion formula was used with the assumption of 95% confidence level, 5% margin of error, 25% estimated prevalence of undernutrition among lactating mothers [19], and 10% nonresponse rate. Then the total sample size was 414. To use a simple random sampling technique, a sampling frame was prepared by reviewing the health posts' family folders and records from all 20 kebeles of the district. To follow a simple random sampling technique, a computer-generated random number was used to select the mothers.

### 2.4. Data Collection Tools and Procedure

Data was collected through a home-to-home visit using a structured and pretested interviewer-administered paper-based questionnaire and measurements. Socioeconomic and demographic characteristics, antenatal care (ANC) utilization, gravidity, maternal and child feeding practice, environmental health condition of the household, household food security, and wealth status were the components of the questionnaire. Household food insecurity access scale (HFIAS) was used to assess the food security status of the households, which was developed by the Food and Nutrition Technical Assistance [20]. The mother's dietary diversity was measured by the recall of all food consumed by the mother during the previous 24 hours, which is according to the Food and Agricultural Organization's (FAO's) guideline for measuring household and individual dietary diversity [21]. Nutrition knowledge was measured using questions that are developed for assessing the practical nutrition knowledge of the lactating mothers. Wealth index questions were obtained from the Ethiopia Demographic and Health Survey, which was based on the household ownership of the productive asset and household characteristics [6]. In addition, the weight and height of the mothers were measured. The weight of the mothers was measured to the nearest 0.1 kg on a battery-powered digital scale. The height of the mothers was measured to the nearest 0.1 cm using a wooden height-measuring board with a sliding head bar following standard anthropometric techniques [22].

### 2.5. Data Quality Control

The questionnaire was initially developed in English and translated into Kambatic (local) language. Residents who completed high school and were fluent in speaking and writing of Kambatic language were recruited for data collection, and nurses were recruited for supervision. The training was given for two days by the investigator. The questionnaire was retranslated back to English by an individual who was blind to the original English version for checking consistency. Pretest of the questionnaire was employed prior to the actual data collection period on 5% of the sample size, and modification was made based on the finding. The functionality of digital weight scales was checked using known weight every morning before data collection began, and the data collectors were assured that the scale reading is exactly at zero before every weight measurement. Supervision was done by the investigator and supervisors, and they checked the collected data for completeness, accuracy, and consistency throughout the data collection period.

### 2.6. Variables

The independent variables include sociodemographic characteristics of the mother and head of the household, family size, source of water for the household, dietary diversity of the mother, frequency of breastfeeding, age of introduction of complementary food for the child and its frequency, cultural avoided food, and nutritional knowledge of the mother. After generating the number of diversified food types consumed by the mothers using FAO's manual [21], the dietary diversity score was calculated and the mean score (4.5 food groups) was used to classify low and high diversity. Nutrition knowledge index categories were generated based on the proportion of questions correctly responded to by the three groups. In addition, household food insecure level and wealth index were also included. The HFIAS category was calculated, and households' food security status was categorized into four based on the Food and Nutrition Technical Assistance manual [20]. Wealth index was generated using a principal component factor analysis based on the household ownership of the productive asset and household characteristics and categorized into three quantiles [6]. The dependent variable is body mass index (BMI) which was categorized as undernutrition for those with less than 18.5 kg/m^2^.

### 2.7. Data Analysis

All the questionnaires were checked visually, coded, and entered into Epi info version 3.5.4 and imported to SPSS Version 20.0 software package for analysis. To assess the presence of an association between dependent and independent variables, bivariate analysis was done. Variables with a *p* value of less than 0.20 were entered into multiple logistic regression model to identify the independent predictors of undernutrition of lactating mothers. The presence of an association between independent and dependent variables using multiple logistic regression model was assessed by using an odds ratio of a *p* value of less than 0.05. For the assessment of multicollinearity, variable inflation factors were used. The fitness of the model was tested by Hosmer–Lemeshow goodness of fit test and the test revealed a *p* value of 0.289.

### 2.8. Ethical Approval and Consent to Participate

Ethical approval for the study was obtained from Institutional Review Board of Arba Minch University (ሕጤሳኮ/4284/54; Date 11/02/2009 EC). Permission to conduct the study was obtained from Angecha District Health Office. Verbal informed consent was obtained from each study participant before the interviews and measurement. The privacy of the study participants was maintained by interviewing the mother alone. The interview was conducted by the data collector alone and sometimes with the presence of the supervisor.

## 3. Results

### 3.1. Sociodemographic Characteristics of Lactating Mothers

Four hundred and ten lactating mothers participated in the study and the response rate was 99.0%. The mean (SD) age of study participants was 27.4 (5.2) years. Of the participants, the majority were from the Kembata ethnic group (82.0%), rural residents (80.5%), married (96.3%), and housewives (80.98%). One hundred seventy-eight (43.4%) participants attended formal education (Table 1).

The mean (SD) family size was 5.0 (1.6) individuals. Based on the HFIAS classification, 23 (5.6%) of the study participants' households were in the severe food insecure category. Most of the participants' households (89.0%) were headed by the husband. Nearly half of the husbands' occupations were farming (57.3%) and attended formal education (50.2%). Almost all (98. 8%) of the households used protected water sources for drinking (Table 2).

### 3.2. Reproductive Health- and Nutrition-Related Factors

From the total study participants, 242 (59.0%) experienced pregnancy 3 or 4 times and 313 (76.3%) received ANC at least once during their last pregnancy. Three hundred sixty-two (88.3%) lactating women introduced complementary feeding but only 143 (39.5%) were started at 6 months (at the recommended age). The mean dietary diversity score (SD) of study participants was 4.5 (1.6) with a range from 1 to 8. The mean (SD) age of breastfeeding children was 13.4 (5.4) months, and more than half (54.9%) were 12 and above months of age (Table 3).

### 3.3. Prevalence of Undernutrition among Lactating Mothers

Of the total lactating mothers included in the study, 21.2% (95% CI: 17.52, 25.46) were undernourished (BMI < 18.5 kg/m^2^). Overweight (BMI > 25 kg/m^2^) was 10.5% (95% CI: 7.86, 13.86). The mean (SD) BMI of the mothers was 21.39 (2.80) kg/m^2^.

### 3.4. Factors Associated with Undernutrition of Lactating Mothers

Based on bivariate analysis, the age of the mothers, gravidity, ANC follow-up, dietary diversity of the mothers, residency, household food security, and wealth index were significantly associated with the undernutrition of lactating mothers. In multivariable logistic regression analysis, the age of mothers, ANC visits, dietary diversity score, and wealth index were significantly associated with the undernutrition of lactating mothers in the study area. Lactating mothers within the age group of 17–25 were four (AOR 4.12, 95% CI: 1.25–13.63) times more likely to be undernourished compared to those mothers in the age group of 36–49 years. The frequency of ANC visits had a significant association with the nutritional status of the lactating mothers. Mothers who had no ANC visits had nearly 3 times (95% CI: 1.43–5.86) higher odds to be malnourished than those who had more than 4 ANC visits. Regarding the dietary diversity score of lactating mothers, those mothers whose dietary diversity score less than the mean (4.5 food groups) were 2.4 (95% CI: 1.35–4.36) times more likely undernourished than those who had to consume five or more food groups. In this study, the family wealth index is another factor that was significantly associated with undernutrition among lactating mothers. The odds of undernutrition among lactating women from 3^rd^ quantile wealth index households reduced by nearly half (AOR 0.47, 95% CI: 0.23–0.98) compared to lactating mothers from 1^st^ quantile wealth index households (Table 4).

## 4. Discussion

The study showed that the prevalence of undernutrition among lactating mothers was 21.2%. Studies conducted in Adama District, Ambo District, Nekemte town, and Raya District of Ethiopia showed the prevalence of undernutrition among lactating mothers from 19.5% to 21.5%, which is similar to this report [23–26]. In addition, the prevalence of undernutrition among lactating mothers was slightly lower than studies conducted in Northern Ethiopia (Womberma and Samre Districts) which was nearly around 25% [19, 27]. Compared to this finding, a significantly higher prevalence of undernutrition among lactating mothers was reported by different studies conducted in Ethiopia. For instance, studies conducted at Kilte Awaleo-Health and Demographic Surveillance Site, Dedo, Seqa-chekorsa, Babile, Endreta, and Hintalo Wajirat Districts of Ethiopia, showed that the ranges of the prevalence of undernutrition among lactating mothers were from 38% to 54.7% [28–30]. The nutritional baseline survey report by Ethiopian Health and Nutritional Research Institute showed the prevalence was 28.8% [31]. On the contrary, the prevalence of this finding is much higher than studies conducted in Kenya (10%) and Nigeria (5%) [32, 33].

Mothers within the age group of 17–25 years (younger) had nearly 4 times higher odds of undernutrition compared with mothers within the age group of 36–49 years (older). This is in line with a result from Ethiopia Health and Nutrition Research Institute, which indicated that younger women were more likely to be malnourished [31]. In addition, studies conducted at Womberma and Ambo Districts of Ethiopia showed similar pattern regarding the increase of the prevalence of undernutrition among young lactating women [26, 27]. Even if the relationship was significant only for lowlanders, a comparative cross-sectional study conducted at Raya Alamata District, Northern Ethiopia, showed that a higher proportion of malnourished women was observed in the younger lactating mothers [23]. A report by Bhandari et al. on women of reproductive age group based on demographic survey data of Nepal prevailed that younger women were more likely to be malnourished than older women [34]. This might be related to the increased nutritional needs of younger women and additional requirements imposed due to prior pregnancy and recent lactation [35]. On top of the physiological vulnerability, inadequate use of maternal health services like ANC and skilled birth attendance among young mothers may contribute to the increment of the prevalence of undernutrition [36]. Another study conducted in Jimma Zone, Southwest Ethiopia, showed that undernutrition was not associated with the age of the mothers [28].

On the other hand, women who had not visited ANC had nearly 3 times higher odds of undernutrition compared to those who had frequent (four or more) ANC visits during the last pregnancy. This result is in line with the study conducted in Samara District, Northern Ethiopia [19]. Repeated visits to the health facility during the ANC follow-up increase the exposure of the mothers to health and nutritional education and other health facility-based interventions, which might reduce the riskiness of the mother for malnutrition.

The dietary diversity score of mothers was found to be associated with undernutrition. Study participants who ate less than five food groups on the preceding day of the interview were more likely to be undernourished. A study conducted in Jimma Zone, Southwest Ethiopia, and Moyale District, Borena Zone, Southern Ethiopia, on lactating mothers showed that the dietary diversity score of the mother was associated with undernutrition [31, 37]. Among lactating mothers, dietary monotony is commonly associated with lower socioeconomic status, unemployment, food insecurity, and illiteracy [38–40]. A study conducted among women of Burkina Faso showed that dietary score is a proxy for overall dietary quality and is also linked with the nutritional status of women [41].

The household wealth status is crucial to improve the nutritional status of lactating mothers. The current study finding also supports this evidence. The odds of mothers from higher wealth index (richer) households were nearly lower by half to develop undernutrition compared to those from lower wealth index (poor) households. A study at Ambo District, Ethiopia, showed that lactating mothers from poor households were 1.76 times more likely to be undernourished than their counterparts (richer) [26]. A report based on demographic and health surveys in Burkina Faso and the Congo Democratic Republic on married women of reproductive age group supports the finding of this research. It indicated that women from poor households were more likely to be undernourished than from the rich [42, 43]. This might be related to the association of poverty, which was indicated as a lower wealth index, and the worsening of a range of key human attributes, including health and nutritional status.

Recall and social desirability biases could be the limitations of the study. For categorizing women to undernutrition, we used a BMI of less than 18.5 kg/m^2^. As it is difficult to obtain a cutoff point for lactating mothers and due to the physiological increase in body weight during pregnancy, which also remains during lactating, it may contribute to the lower prevalence of undernutrition. Even if we excluded known pregnant lactating mothers, in this study we did not use biomarkers for the assessment of pregnancy. It is also difficult to know the usual dietary diversity using a single-day assessment.

## 5. Conclusions

Lactating mothers' undernutrition is prominent in this study setting. One in five lactating mothers was undernourished. Younger age group, not receiving ANC, consuming less diversified food, and from the low wealth status households were associated with undernutrition of lactating mothers. Therefore, due attention should have to be given to younger lactating mothers and to increase the ANC attendance of the women. In addition, alternative approaches should have to be required for households with poor socioeconomic status and to increase the dietary diversity of mothers.

## Figures and Tables

**Table 1:** Sociodemographic characteristics of lactating mothers (*N* = 410).

Variables	Categories	Undernutrition		Total
		Yes *N* (%)	No *N* (%)	
Age of mothers	17–25 years	56 (31.1)	124 (68.9)	180 (43.9)
	26–35 years	27 (13.6)	171 (86.4)	198 (48.3)
	36–49 years	4 (12.5)	28 (87.5)	32 (7.8)

Marital status	Married	83 (21.0)	312 (79.0)	395 (96.3)
	Others^*∗*^	4 (26.7)	11 (73.3)	15 (3.7)

Ethnicity	Kambata	67 (19.9)	269 (80.1)	336 (82.0)
	Hadiya	18 (33.3)	36 (66.7)	54 (13.2)
	Other	2 (10.0)	18 (90.0)	20 (4.9)

Religion	Protestant	65 (22.8)	220 (77.2)	285 (69.5)
	Orthodox	9 (14.8)	52 (85.3)	61 (14.9)
	Adventist	7 (16.7)	35 (83.3)	42 (10.2)
	Other	6 (27.3)	16 (72.7)	22 (5.4)

Residence	Rural	79 (23.9)	251 (76.1)	330 (80.5)
	Urban	8 (10.0)	72 (90.0)	80 (19.5)

Mothers' occupation	Farmer	10 (20.41)	39 (79.6)	49 (12.0)
	Housewife	71 (23.4)	233 (76.6)	304 (74.2)
	Gov. employment	2 (7.1)	26 (92.9)	28 (6.8)
	Other	4 (13.8)	25 (86.2)	29 (7.1)

Educational status of mothers	Unable to read and write	26 (21.1)	97 (78.9)	123 (30.0)
	Read and write only	21 (19.3)	88 (80.7)	109 (26.6)
	Formal education	40 (22.5)	138 (77.5)	178 (43.4)

^*∗*^Include never married and divorced.

**Table 2:** Characteristics of the households of lactating mothers (*N* = 410).

Variables	Categories	Undernutrition		Total
		Yes *N* (%)	No *N* (%)	
Head of the household	Mother	11 (26.8)	30 (73.2)	41 (10.0)
	Husband	74 (20.4)	291 (79.7)	365 (89.0)
	Other^*∗*^	2 (50.0)	2 (50.0)	4 (1.0)
Educational status of head of the household	Unable to read and write	21 (24.7)	64 (75.3)	85 (20.7)
	Able to read and write	25 (21.0)	94 (79.0)	119 (29.0)
	Formal education	41 (19.9)	165 (80.1)	206 (50.2)
Occupation of head of the household	Farmer	51 (21.7)	184 (78.3)	235 (57.3)
	Gov. employment	8 (11.0)	65 (89.0)	73 (17.8)
	Private labor	18 (29.5)	43 (70.5)	61 (14.9)
	Daily laborer	10 (24.4)	31 (75.6)	41 (10.0)
Number of children of age <5 years	One	80 (22.3)	279 (77.7)	359 (87.6)
	Two	7 (13.7)	44 (86.3)	51 (12.4)
Family member size	1–4	32 (19.4)	133 (80.6)	165 (40.2)
	>4	55 (22.5)	190 (77.6)	245 (59.8)
Household food insecure level	Food secure	69 (19.6)	284 (80.5)	353 (86.1)
	Mild food insecure	7 (25.9)	20 (74.1)	27 (6.6)
	Moderately food insecure	3 (42.9)	4 (57.1)	7 (1.7)
	Severely food insecure	8 (34.8)	15 (65.2)	23 (5.6)
Wealth index	1^st^ quantile	29 (21.2)	108 (78.8)	137 (33.4)
	2^nd^ quantile	39 (28.5)	98 (71.5)	137 (33.4)
	3^rd^ quantile	19 (14.0)	117 (86.0)	136 (33.2)
Source of water	Pipe	66 (20.0)	265 (80.1)	331 (80.7)
	Protected	18 (24.3)	56 (75.7)	74 (18.1)
	Unprotected	3 (60.0)	2 (40.0)	5 (1.2)

^*∗*^Her/his father or mother and grandparents.

**Table 3:** Reproductive health- and nutrition-related factors among lactating mothers (*n* = 410).

Variables	Categories	Undernourished		Total *N* (%)
		Yes *N* (%)	No *N* (%)	
Gravidity	≤2	33 (21.6)	120 (78.4)	153 (37.3)
	3–4	48 (19.8)	194 (80.2)	242 (59.0)
	>4	6 (40.0)	9 (60.0)	15 (3.7)
ANC visit	No ANC	32 (33.0)	65 (67.0)	97 (23.7)
	<4 times	36 (20.3)	141 (79.7)	177 (43.2)
	≥4 times	19 (14.0)	117 (86.0)	136 (33.2)
Age of breastfeeding child	<12	32 (17.3)	153 (82.7)	185 (45.1)
	≥12	55 (24.4)	170 (75.6)	225 (54.9)
Breastfeeding frequency	≤12	18 (23.7)	58 (76.3)	76 (18.5)
	>12	69 (20.7)	265 (79.3)	334 (81.5)
Introduction of complementary feeding	Yes	78 (21.6)	284 (78.5)	362 (88.3)
	No	9 (18. 8)	39 (81.3)	48 (11.7)
Frequency of meals (*N* = 362)	<4	76 (22.1)	268 (77.9)	344 (95.0)
	≥4	2 (11.1)	16 (88.9)	18 (5.0)
Cultural avoided food	Yes	3 (33.3)	6 (66.7)	9 (2.2)
	No	84 (21.0)	317 (79.1)	401 (97.8)
Nutritional knowledge	Poor	12 (35.3)	22 (64.7)	34 (8.3)
	Fair	37 (21.5)	135 (78.5)	172 (42.0)
	Good	38 (18.6)	166 (81.4)	204 (49.8)
Women dietary diversity score	<5	64 (29.9)	150 (70.1)	214 (52.2)
	≥5	23 (11.7)	173 (88.3)	196 (47.8)

**Table 4:** Factors associated with undernutrition in multiple logistic analysis of flactating women (*N* = 410).

Variables	Categories	COR (95% CI)	AOR (95% CI)
Age of mothers	17–25 years	3.16 (1.06, 9.44)^*∗*^	4.12 (1.25, 13.63)^*∗*^
	26–35 years	1.11 (0.36, 3.40)	1.50 (0.45, 4.93)
	36–49 years	1	1

Residence	Rural	2.83 (1.31, 6.14)^*∗*^	2.32 (0.94, 5.74)
	Urban	1	1

ANC category	No ANC	3.03 (1.59, 5.77)^*∗*^	2.90 (1.43, 5.86)^*∗*^
	<4 times	1.57 (0.86, 2.89)	1.45 (0.75, 2.81)
	≥4 times	1	1

Age of breastfeeding child	≤12 months	1	1
	>12 months	1.55 (0.95, 2.52)	1.53 (0.89, 2.63)

Dietary diversity score	<5	3.20 (1.90, 5.42)^*∗*^	2.42 (1.35, 4.36)^*∗*^
	≥5	1	1

HFIAS category	Food secure	1	1
	Food insecure	1.90 (1.02, 3.52)^*∗*^	1.46 (0.74, 2.88)

Gravidity	1	1	1
	2–4	0.81 (0.49, 1.34)	1.65 (0.89, 3.03)
	≥5	4.44 (1.70, 11.62)^*∗*^	2.05 (0.56, 7.45)

Wealth index	1^st^ quantile	1	1
	2^nd^ quantile	1.48 (0.85, 2.58)	0.96 (0.49, 1.88)
	3^rd^ quantile	0.60 (0.32, 1.14)	0.47 (0.23, 0.98)^*∗*^

^*∗*^ *p* value <0.05.

## Data Availability

The data used to support the findings of this study are available from the corresponding author on reasonable request.

## Abbreviations

ANC: Antenatal care
BMI: Body mass index
FAO: Food and Agricultural Organization
HFIAS: Household food insecurity access scale.

## References

[1] World Bank (2006). *Repositioning Nutrition as Central to Development: A Strategy for Large Scale Action*.

[2] Black R. E., Allen L. H., Bhutta Z. A. (2008). Maternal and child undernutrition: global and regional exposures and health consequences. *The Lancet*.

[3] Tang A. M., Chung M., Dong K. (2016). Determining a global mid-upper arm circumference cutoff to assess malnutrition in pregnant women. *Food and Nutrition Technical Assistance*.

[4] World Health Organization (2011). *Nutrition of Women in the Preconception Period, during Pregnancy and the Breastfeeding Period*.

[5] Lartey A. (2008). Maternal and child nutrition in sub-saharan Africa: challenges and interventions. *Proceedings of the Nutrition Society*.

[6] Central Statistical Authority (2011). *Ethiopia Demographic and Health Survey. In.: Addis Ababa, Ethiopia and Calverton*.

[7] World Health Organization (2000). *Nutrition for Health and Development: A Global Agenda for Combating Malnutrition. In: Progress Report WHO/NHD/SDE: 2000*.

[8] United Nations University World Health Organization (2004). *Human Energy Requirements: Report of a Joint FAO/WHO/UNU Expert Consultation: Rome*.

[9] Allen L. H. (2012). B vitamins in breast milk: relative importance of maternal status and intake, and effects on infant status and function. *Advances in Nutrition*.

[10] Briend A., Dewey K. G., Reinhart G. A. (2011). Fatty acid status in early life in low-income countries—overview of the situation, policy and research priorities. *Maternal & Child Nutrition*.

[11] Daniels L., Gibson R. S., Diana A. (2019). Micronutrient intakes of lactating mothers and their association with breast milk concentrations and micronutrient adequacy of exclusively breastfed Indonesian infants. *The American Journal of Clinical Nutrition*.

[12] Branca F., Piwoz E., Schultink W., Sullivan L. M. (2015). Nutrition and health in women, children, and adolescent girls. *BMJ Journal*.

[13] Christian P. (2002). Maternal nutrition, health, and survival. *Nutrition Reviews*.

[14] Food and Agriculture Organization (2002). *Food Insecurity in the World: When People Must Live With Hunger and Fear Starvation. In: 2002: Food and Agriculture Organization of the United Nations*.

[15] Federal Ministry of Health (2008). *National nutrition Strategy of Ethiopia. In. Addis Ababa, Ethiopia*.

[16] Government of Ethiopia (2016). *National Nutrition Program, 2016–2020*.

[17] Federal Democratic Republic of Ethiopia (2016). *Seqota Declaration Implementation Plan (2016–2030): Summary Programme Approach Document*.

[18] Central Statistical Agency (2013). *Population Projection of Ethiopia for all Regions at Wereda Level from 2014–2017. In: Journal of Ethnobiology and Ethnomedicine: 2013: Federal Democratic Republic of Ethiopia*.

[19] Haileslassie K., Mulugeta A., Girma M. (2013). Feeding practices, nutritional status and associated factors of lactating women in Samre Woreda, south eastern zone of Tigray, Ethiopia. *Nutrition Journal*.

[20] Coates J., Swindale A, Bilinsky P. (2007). *Household Food Insecurity Access Scale (HFIAS) for Measurement of Food Access: Indicator Guide*.

[21] Food and Agriculture Organization (2010). *Guidelines for Measuring Household and Individual Dietary Diversity*.

[22] Cogill B. (2003). *Anthropometric Indicators Measurement Guide*.

[23] Sitotaw I. K., Hailesslasie K., Adama Y. (2017). Comparison of nutritional status and associated factors of lactating women between lowland and highland communities of district Raya, Alamata, southern Tigiray, Ethiopia. *BMC Nutrition*.

[24] Hundera T. D., Gemede H. F., Wirtu D., Kenie D. N. (2015). Nutritional status and associated factors among lactating mothers in nekemte referral hospital and health centers, Ethiopia. *Food Science and Quality Management*.

[25] Biru K., Jima A., Abeya S. (2017). Prevalence of chronic energy malnutrition and maternal health service utilizations among lactating mothers in Adama district, Oromia region, eastern Ethiopia. *Journal of Food Processing and Technology*.

[26] Zerihun E., Egata G., Mesfin F. (2016). Under nutrition and its associated factors among lactating mothers in rural Ambo district, west Shewa zone, Oromia region, Ethiopia. *East African Journal of Health and Biomedical Sciences*.

[27] Berihun S., Kassa G. M., Teshome M. (2017). Factors associated with underweight among lactating women in Womberma woreda, northwest Ethiopia; a cross-sectional study. *BMC Nutrition*.

[28] Alemayehu M., Argaw A., Mariam A. G. (2015). Factors associated with malnutrition among lactating women in subsistence farming households from Dedo and seqa-chekorsa districts, Jimma zone, 2014. *Developing Country Studies*.

[29] Abera S. F., Kantelhardt E. J., Bezabih A. M. (2020). What factors are associated with maternal undernutrition in eastern zone of Tigray, Ethiopia? evidence for nutritional well-being of lactating mothers. *BMC Public Health*.

[30] Roba K. T., O’Connor T. P., Belachew T., O’Brien N. M. (2015). Seasonal variation in nutritional status and anemia among lactating mothers in two agro-ecological zones of rural Ethiopia: a longitudinal study. *Nutrition*.

[31] Ethiopian Health and Nutrition Research Institute (2010). *Nutritional Baseline Survey Report for the National Nutrition Program of Ethiopia. In: 2010; Addis Ababa, Ethiopia*.

[32] Gewa C. A., Oguttu M., Yandell N. S. (2012). Maternal nutrition in rural Kenya: health and socio-demographic determinants and its association with child nutrition. *Maternal & Child Nutrition*.

[33] Sanusi R. A, Falana O. A. (2007). The nutritional status of mothers practicing breast feeding in ibadan, Nigeria. *African Journal of Biomedical Research*.

[34] Bhandari S., Sayami J. T., Thapa P., Sayami M., Kandel B. P., Banjara M. R. (2016). Dietary intake patterns and nutritional status of women of reproductive age in Nepal: findings from a health survey. *Archives of Public Health*.

[35] World Health Organization (2006). *Adolescent Nutrition: A Review of the Situation in Selected South-East Asian Countries*.

[36] Singh P., Singh K. K., Singh P. (2021). Maternal health care service utilization among young married women in India, 1992–2016: trends and determinants. *BMC Pregnancy and Childbirth*.

[37] Bekele H., Jima G. H., Regesu A. H. (2020). Undernutrition and associated factors among lactating women: community-based cross-sectional study in Moyale district, Borena zone, southern Ethiopia. *Advances in Public Health*.

[38] Singh D. R., Ghimire S., Upadhayay S. R., Singh S., Ghimire U. (2020). Food insecurity and dietary diversity among lactating mothers in the urban municipality in the mountains of Nepal. *PLoS One*.

[39] Boke M. M., Geremew A. B. (2018). Low dietary diversity and associated factors among lactating mothers in Angecha districts, southern Ethiopia: community based cross-sectional study. *BMC Research Notes*.

[40] Weldehaweria N. B., Misgina K. H., Weldu M. G. (2016). Dietary diversity and related factors among lactating women visiting public health facilities in Aksum town, Tigray, northern Ethiopia. *BMC Nutrition*.

[41] Savy M., Martin-Prével Y., Sawadogo P., Kameli Y., Delpeuch F. (2005). Use of variety/diversity scores for diet quality measurement: relation with nutritional status of women in a rural area in Burkina Faso. *European Journal of Clinical Nutrition*.

[42] Adebowale A., Palamuleni M., Odimegwu C. (2015). Wealth and under-nourishment among married women in two impoverished nations: evidence from Burkina Faso and Congo democratic republic. *BMC Research Notes*.

[43] Savy M., Martin-Prével Y., Danel P., Traissac P., Dabiré H., Delpeuch F. (2008). Are dietary diversity scores related to the socio-economic and anthropometric status of women living in an urban area in Burkina Faso?. *Public Health Nutrition*.

